# Accurate single nucleotide variant detection in viral populations by combining probabilistic clustering with a statistical test of strand bias

**DOI:** 10.1186/1471-2164-14-501

**Published:** 2013-07-24

**Authors:** Kerensa McElroy, Osvaldo Zagordi, Rowena Bull, Fabio Luciani, Niko Beerenwinkel

**Affiliations:** 1 Centre for Marine Bioinnovation and School of Biotechnology and Biomolecular Sciences, University of New South Wales, Sydney, NSW, Australia; 2 Inflammation and Infection Research Group, Evolutionary Dynamics of Infectious Diseases, School of Medical Sciences, Sydney, NSW, Australia; 3 Institute for Medical Virology, University of Zurich, Zurich, Switzerland; 4 Department of Biosystems Science and Engineering, ETH Zurich, Basel, Switzerland; 5 SIB Swiss Institute of Bioinformatics, Basel, Switzerland

## Abstract

**Background:**

Deep sequencing is a powerful tool for assessing viral genetic diversity. Such experiments harness the high coverage afforded by next generation sequencing protocols by treating sequencing reads as a population sample. Distinguishing true single nucleotide variants (SNVs) from sequencing errors remains challenging, however. Current protocols are characterised by high false positive rates, with results requiring time consuming manual checking.

**Results:**

By statistical modelling, we show that if multiple variant sites are considered at once, SNVs can be called reliably from high coverage viral deep sequencing data at frequencies lower than the error rate of the sequencing technology, and that SNV calling accuracy increases as true sequence diversity within a read length increases. We demonstrate these findings on two control data sets, showing that SNV detection is more reliable on a high diversity human immunodeficiency virus sample as compared to a moderate diversity sample of hepatitis C virus. Finally, we show that in situations where probabilistic clustering retains false positive SNVs (for instance due to insufficient sample diversity or systematic errors), applying a strand bias test based on a beta-binomial model of forward read distribution can improve precision, with negligible cost to true positive recall.

**Conclusions:**

By combining probabilistic clustering (implemented in the program ShoRAH) with a statistical test of strand bias, SNVs may be called from deeply sequenced viral populations with high accuracy.

## Background

Next generation sequencing technologies have unprecedented potential for assessing genetic diversity in viral populations [1,2]. As compared to traditional Sanger bulk sequencing, the cost per base of sequencing is low, due in part to the massively parallel nature of these technologies [3]. Roche-454 is currently the most popular technology to investigate diversity in viral populations, thanks to its superior read length compared to other platforms such as Illumina GAIIx [4], but both technologies have advantages and disadvantages for analysing viral diversity, suggesting that experimental design should be planned carefully [5]. Currently, one run of Roche-454 sequencing can produce one million reads, with a modal read length of 700nt - enough to sequence five 10kb viral genomes to a coverage of 20,000x each. This high coverage facilitates deep sequencing experiments, where reads are treated as a population sample containing information about the underlying genetic structure of the population. For example, deep sequencing has been used to study bottlenecks in early HCV infection [6] and to identify mutations associated with drug resistance in HIV [7,8].

A major challenge in deep sequencing experiments involves separating true variants from sequencing errors. Although average error rates are constantly improving, error rates remain highly heterogeneous as they are affected by sequence context (for instance, homopolymer sequences) and alignment errors [9,10]. This means that locally, error rates can still be quite high, leading to false positive SNV calls [11].

A number of tools are available for calling SNVs within the context of sequencing errors. However, most approaches are tailored to calling SNVs in human resequencing projects and are not suitable for viral deep sequencing data. When resequencing a human genome, SNVs are either heterologous and can be expected to be present in 50% of all reads, or homologous and should be present in 100% of reads. Commonly, a probabilistic approach is employed to decide whether a potential SNV is either an error, a homologous SNV, or a heterologous SNV [12,13]. In deep sequencing experiments, however, very low frequency SNVs, including SNVs present in less than 1% of reads, are often of interest [4,14]. For instance, the presence of low frequency variants in the viral population of a single host may determine the potential for drug resistance and treatment failure [7,15].

To date, deep sequencing data has been analysed by applying various read quality filters before and after alignment and then further manually filtering the results, according to the researcher’s understanding of the sequencing technology and the individual data set [16]. Filtering may also be performed with a software program such as VarScan [17], an SNV caller which relies on read coverage, base qualities, and variant frequencies to separate true SNVs from errors. An emerging statistical method for detecting SNVs in deep sequencing data involves performing a statistical test for each potential SNV, taking into account a prior positional error model [18]. This method detected SNVs with a population frequency of 0.1% in control Illumina data. However, the requirement for a prior positional error model relies on the availability of multiple replicates, which is not always feasible.

A more recent approach to SNV detection in viral populations, implemented in the program V-Phaser, utilises the covariance between observed SNVs present on the same read to increase sensitivity [19]. The power of covariance was also harnessed in a recent study of HCV early evolution, where SNV calling was achieved by parsing haplotypes reconstructed using probabilistic clustering performed by the program ShoRAH [6]. ShoRAH uses a Bayesian approach to group reads into haplotypes. Both sensitivity and specificity are improved by assuming that the consensus sequence for each cluster is the true haplotype sequence; all other variants present in the reads within a cluster are considered to be errors [20,21]. ShoRAH has been shown to accurately reconstruct variants with a population frequency of 0.1%, and to efficiently remove random PCR errors [22]. Error correction via probabilistic clustering will fail, however, when errors themselves appear clustered. This can occur in regions of low diversity characterised by an absence of true variants, or when sequencing errors are systematic (e.g., in homopolymeric regions). The potential for systematic errors to confound clustering may explain why an empirical k-mer based algorithmic approach was found to be more efficient than ShoRAH at removing false haplotypes, although both approaches detected all true haplotypes [23].

There is growing evidence that systematic errors may exhibit a strand bias, where a particular error is more likely to occur in reads traversing the genome in one direction as opposed to the other [24]. For instance, in a recent analysis addressing errors from control Illumina data using prior positional error models, error rates between forward and reverse reads were uncorrelated [18]. Because of this, several SNV detection methods consider forward reads and reverse reads separately, using some combination of the two obtained p-values to inform the final SNV calling decision [18,25]. Researchers have also used strand bias directly to filter out false positive SNVs. For example, in a deep sequencing analysis of HIV-1 quasispecies, any potential SNVs with more than 10 fold difference in the forward and reverse read count were considered to be systematic errors [16], while in the HCV study mentioned above, raw results from ShoRAH were further cleaned by removing any potential SNVs that showed a difference of more than 10% between the number of forward and reverse reads [6]. The latest version of Samtools formalises this approach, reporting a p-value based on an exact test of strand bias [26]. A two tailed Fisher’s exact test is also included in the recently released SNV caller LoFreq [27].

Here, we show that in addition to its primary function of haplotype reconstruction, ShoRAH may also be used to reliably call SNVs. This is demonstrated through analysis of control Roche-454 data from both HCV and HIV samples, and by development of a statistical argument in support of SNV calling via probabilistic clustering. As strand bias has been observed in systematic errors retained during clustering [6], we also investigate the benefits of combining probabilistic clustering with a statistical test of strand bias.

## Results

**Figure 1:**
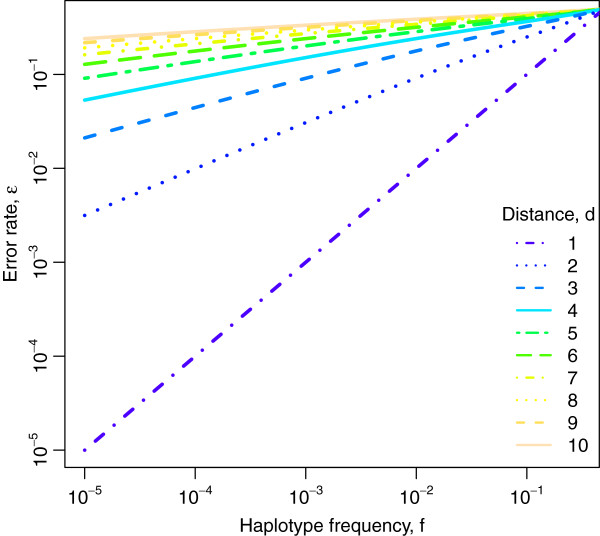
**Limit of SNV detection.** Shown is the maximal error rate *ϵ* at which SNVs of frequency *f* are detectable if *d* sites are analysed simultaneously. In a simple model of SNV calling (see Methods), this bound is *ϵ*^*d*^ < 1/[1 + (1 − *f*)/*f*] ≈ *f*, giving rise to the lines of slope 1*/d* for two haplotypes at Hamming distances *d* in the double logarithmic plot.

Our SNV calling method is based on two assumptions: (1) it is more powerful to compare several variants that co-occur on the same read simultaneously than individually; and (2) the frequency of sequencing errors, but not of true SNVs, is often higher on reads sequenced in one direction than the other. While there is empirical evidence for the second assumption, the first one can be motivated and made precise by a simplified statistical model of SNV detection (see Methods). In this model, two haplotypes of frequencies *f* and 1 − *f* that differ at *d* sites co-occurring within an observed read, can be distinguished in the presence of a per site error probability *ϵ* only if (approximately) *f > ϵ*^*d*^. For *d* = 1, this result confirms the intuitive notion that a single SNV can be detected only if the error rate is smaller than the SNV frequency. However, if the haplotypes differ at two sites (*d* = 2), then the probability that these two variants are both errors is *ϵ*^2^, and the limit of haplotype detection decreases to *f > ϵ*^2^. In general, considering multiple variant sites at once by comparing haplotypes increases SNV calling accuracy (Figure 1). This finding motivates analysing entire reads or large segments of reads in order to include as many SNVs as possible, as is done in read clustering methods such as ShoRAH.

To investigate real life performance of SNV calling via probabilistic clustering, we analysed Roche-454 data from two distinct control experiments. For the first experiment, the same library of cloned moderate-diversity HCV fragments mixed at equal quantities was subjected to two independent Roche-454 sequencing runs (HCV run 1 and HCV run 2) [6]. For the second experiment, one round of Roche-454 sequencing was performed on a high diversity PCR amplified set of HIV fragments mixed according to a geometric series [22].

SNVs were called using either probabilistic clustering (ShoRAH), quality score based filtering (VarScan), or a per site quality score based statistical test (LoFreq) [27]. A statistical test of strand bias was applied to the results from ShoRAH and VarScan, utilising either a beta-binomial (or binomial) model of forward read distribution for true SNVs, or Fisher’s exact test (LoFreq includes its own Fisher’s exact test of strand bias). Our experimental design allowed us to assess the performance of probabilistic clustering, and variation in the true SNV strand bias distribution, both between samples with different diversity levels and between individual sequencing runs.

### Alignment and clone frequencies

For HCV run 1 and HCV run 2, 97.1% and 95.7% of reads aligned uniquely to the reference, giving an average read depth of 2564 reads and 3291 reads, respectively. For the HIV data, 88.9% of reads aligned, giving an average read depth of 6915 reads. True haplotype frequency estimates are given in Table 1 (HCV) and Table 2 (HIV). For all samples, the frequencies of at least some haplotypes differed substantially from their intended frequencies. As the estimated frequencies for HCV run 1 and HCV run 2 are very similar, it is likely that deviation from intended frequencies is a result of mixing errors, rather than bias introduced during library preparation and sequencing. Lists of true SNVs for both HCV and HIV samples are provided in the Additional files 1 and 2.

**Table 1:** Estimated true and intended clone frequencies, HCV

**Clone**	**Intended (%)**	**True, run 1 (%)**	**True, run 2 (%)**
023-180,609-2	25.0	37.1	39.2
023-180,609-1	25.0	25.9	24.7
023-180,609-6	25.0	25.0	24.3
023-180,609-5	25.0	12.0	12.0

**Table 2:** Estimated true and intended clone frequencies, HIV

**Clone**	**Intended (%)**	**True (%)**
07-56,951	25	37.6
07-54,825	6.3	33.8
08-04,134	3.1	12.2
08-59,712	12.5	9.1
07-56,681	50	5.6
08-02,659	0.2	0.8
08-01,315	1.6	0.4
08-04,512	0.1	0.2
08-55,163	0.8	0.2
08-57,881	0.4	0.2

### Raw SNV calls

For HCV run 1 and HCV run 2, both ShoRAH and VarScan detected all true SNVs, i.e. the false negative rate was zero (Table 3), while LoFreq called all but one true SNV in each run. VarScan, however, also called up to 206 (run 1) and 826 (run 2) false positives, more than seven times as many as ShoRAH. More errors were incorrectly identified as SNVs for HCV run 2 compared to HCV run 1 by both VarScan and ShoRAH, indicating a higher error rate for HCV run 2. VarScan’s recall was greater than ShoRAH’s for the HIV data, with 93% of true SNVs identified, compared to 81% for ShoRAH. However, ShoRAH’s recall was greater than LoFreq’s, which only identified 64% of true SNVs. 

**Figure 2:**
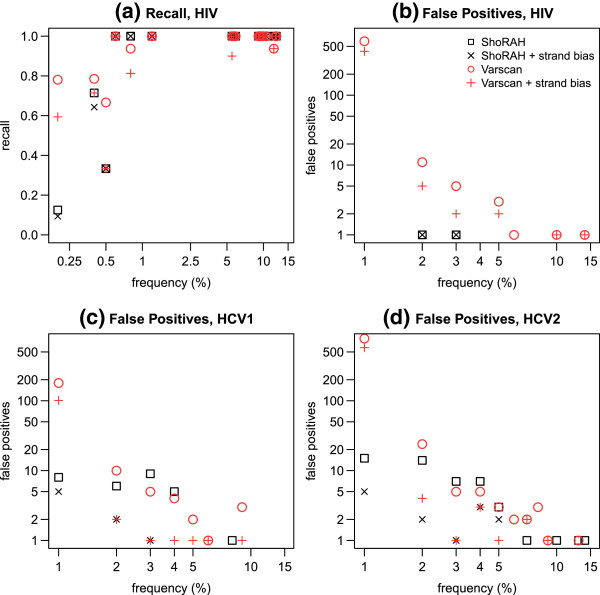
**SNV recall and false positives by frequency.** Detailed analysis of SNV calling for variants with population frequency less than 15%. Both raw results, and results subjected to a strand bias test with *σ*=0.0111, are given. In cases where cross hairs fill a symbol of the same colour, the strand bias test had no effect on results. **(a)** Recall of true SNVs for HIV data, by frequency (calculated using known haplotype frequencies (see Table 2)). Note: for SNVs with population frequency greater than 15%, recall was perfect for all methods. For this reason, recall plots for HCV1 and HCV2 are also omitted, as they are straight lines with perfect recall = 1. **(b)** HIV, **(c)** HCV run 1, and **(d)** HCV run 2, absolute false positive SNV counts by predicted frequency for each method, binned in intervals of 1%. A double logarithmic scale is used for plots **(b)**, **(c)**, and **(d)**; thus data points where no false positives are recorded are omitted. For all ShoRAH runs, no false positives were observed with population frequency greater than 15%. Some false positives with population frequency greater than 15% occurred for the VarScan runs, however close inspection revealed the majority of these to be the result of a VarScan bug when calling SNVs for genomic positions with two or more variants.

For the HIV sample, all of ShoRAH’s false negatives had true population frequencies under 0.5%, while one of VarScan’s false negatives had a true population frequency of 11.9% (see also Figure 2a: recall values less than one indicate false negatives). Upon inspection of the alignment and the VarScan file, this appeared to be the result of confusion in reporting positions with more than one variant allele. Most of LoFreq’s false negatives were very low frequency (under 1%), with three having true population frequencies of under 6%. VarScan’s superior recall was accompanied by a high number of false positive calls – over the reference 766bp considered, 732 false positive SNVs were also called. By comparison, ShoRAH only made two false positive calls (Table 3 and Figure 2b). Overall, LoFreq exhibited an exceptionally low false positive rate. For the HCV runs, one unique false positive was identified in each run, while no false positives were called by LoFreq for the HIV data.

**Table 3:** SNV calling accuracy

**Data set**	**Method**	**ShoRAH**					**VarScan**					**LoFreq**				
		**TP**	**FP**	**FN**	**Rc.**	**Pr.**	**TP**	**FP**	**FN**	**Rc.**	**Pr.**	**TP**	**FP**	**FN**	**Rc.**	**Pr.**
HCV1	Raw	38	29	0	1.000	0.567	38	206	0	1.000	0.156	37	1	1	0.974	0.974
	Fisher’s exact	32	6	6	0.842	0.842	36	179	2	0.947	0.167	29	0	9	0.763	1.000
	Bin. (σ = 0)	37	7	1	0.974	0.841	36	108	2	0.947	0.250					
	B-bin., σ = 0.0004	37	7	1	0.974	0.841	37	108	1	0.947	0.255					
	B-bin., σ = 0.0014	37	8	1	0.974	0.822	37	108	1	0.947	0.255					
	B-bin., σ = 0.0111	38	8	0	1.000	0.826	38	108	0	1.000	0.261					
HCV2	Raw	38	50	0	1.000	0.432	38	826	0	1.000	0.044	37	1	1	0.974	0.974
	Fisher’s exact	33	9	5	0.868	0.785	36	766	2	0.947	0.045	29	0	9	0.763	1.000
	Bin. (σ = 0)	34	8	4	0.895	0.810	35	577	3	0.921	0.057					
	B-bin., σ = 0.0004	36	8	2	0.947	0.818	36	577	2	0.947	0.059					
	B-bin., σ = 0.0014	36	8	2	0.947	0.818	36	577	2	0.947	0.059					
	B-bin., σ = 0.0111	38	13	0	1.000	0.745	38	589	0	1.000	0.061					
HCV2	Raw	153	2	35	0.814	0.987	175	732	13	0.931	0.193	121	0	67	0.644	1.000
	Fisher’s exact	87	0	101	0.462	1.000	125	671	63	0.665	0.157	41	0	147	0.218	1.000
	Bin. (σ = 0)	88	0	100	0.468	1.000	113	473	75	0.601	0.193					
	B-bin., σ = 0.0004	101	1	87	0.537	0.990	121	474	67	0.644	0.203					
	B-bin., σ = 0.0014	126	1	62	0.670	0.992	135	475	53	0.718	0.221					
	B-bin., σ = 0.0111	151	2	37	0.803	0.987	162	490	26	0.862	0.248					

SNV calling statistics for ShoRAH, VarScan, and LoFreq. For ShoRAH and VarScan, SNV calls without the strand bias test (Raw) and using different values of the beta-binomial dispersion parameter *σ* in the strand bias test are given, with *σ* = 0 corresponding to a binomial forward read distribution. For LoFreq, the results of applying our strand bias test are absent as this software does not report forward and reverse strand counts. For all SNV calling methods, the results of applying Fisher’s exact test to the raw output are also given (this was possible for LoFreq as it reports a strand bias Fisher’s exact test p-value for each variant). Reported statistics include true positives (TP), i.e., genomic sites with a variant matching a known true variant, false positives (FP), i.e., genomic sites with a variant that is not a known true variant, and false negatives (FN), i.e., known variants which are not identified by the relevant SNV calling method. Individual genomic sites may contribute to both true positives and false positives. Recall (TP/(TP + FN)) and precision (TP/(TP + FP)) are also reported.

### Strand bias test

In an attempt to improve ShoRAH’s performance under conditions of sub-optimal diversity or clustered systematic errors, we applied a statistical test of strand bias to the raw SNV calls.

For each potential SNV detected, we test the hypothesis that the distribution of forward reads carrying this SNV follows a beta-binomial distribution, with mean value equal to the overall forward read distribution at that position. The overdispersion of the forward read distribution is controlled by the parameter *σ*, which we estimate separately for each sequencing run using maximum likelihood (ML) methods based on the observed forward read distribution for all true SNVs within the sample (see Methods).

ML estimates of *σ* for HCV run 1, HCV run 2, and HIV samples were 0.0004, 0.0014, and 0.0111 respectively. To avoid overfitting, we applied tests using all estimated values of *σ* to each dataset, also performing a test with *σ*=0 (corresponding to a binomial forward read distribution). For comparison with the strand bias test implemented in other software, we also performed a Fisher’s exact test of strand bias.

SNV calling statistics for strand bias tests applied to the raw results of both ShoRAH and VarScan are given in Table 3. The results of LoFreq’s own implementation of Fisher’s exact test are also given. Applying a strand bias test to the raw SNV calls for both ShoRAH and VarScan improved precision in all cases, except for HIV SNV calls made using ShoRAH with *σ* = 0.0111, where precision remained unaltered compared to the raw ShoRAH output. In general, choice of *σ* did not have a substantial impact on precision. However, smaller values of *σ* were more likely to result in reduced recall, as was Fisher’s exact test. Recall was also compromised by LoFreq’s implementation of Fisher’s exact test. This was particularly evident for the HIV data, and is consistent with an overdispersed forward read distribution for true SNVs, justifying our choice of a beta-binomial forward read distribution.

**Figure 3:**
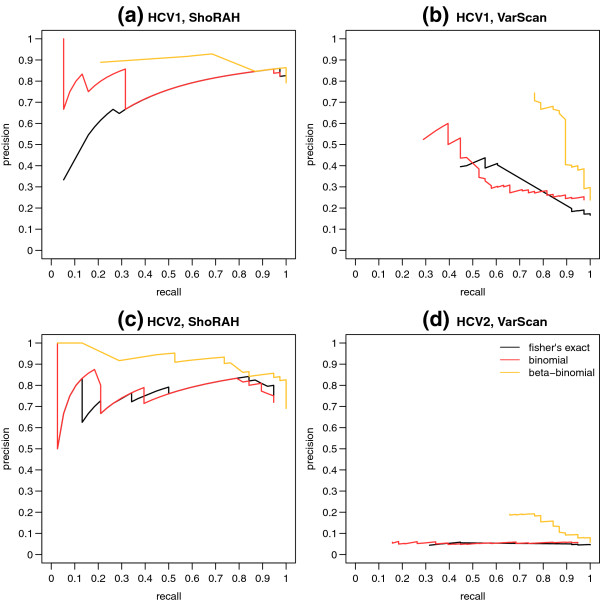
**Precision recall curves for various strand bias tests.** Precision versus recall, for strand bias tests performed on raw SNV calls from **(a)** ShoRAH applied to HCV run 1, **(b)** VarScan applied to HCV run 1, **(c)** ShoRAH applied to HCV run 2, and **(d)** VarScan applied to HCV run 2. In all cases, strand bias tests based on an underlying beta-binomial forward read distribution exhibited greater or equal precision for the same recall value, when compared to either a strand bias test with a binomial forward read distribution, or a Fisher’s exact test of strand bias.

For the beta-binomial test, the reduction in recall was minimised in all cases by using *σ* = 0.0111. To further investigate the appropriateness of a beta-binomial based strand bias test (with *σ* = 0.0111), compared to either a binomial based test or Fisher’s exact test, we generated precision recall curves for each test applied to SNV calls from ShoRAH and VarScan (Figure 3). In all cases, across the full range of recall values obtained the beta-binomial test gave equivalent or higher precision than either of the other tests, confirming its suitability.

For the HCV samples, the strand bias test was particularly good at reducing errors in results from ShoRAH, with more than 70% of errors eliminated. After the strand bias test, less than six errors for every 1000 reference sites remained for HCV run 2, while less than four errors for every 1000 reference sites remained for HCV run 1. The strand bias test also eliminated 48% of errors in VarScan’s output, however as VarScan’s raw output contained more errors than ShoRAH, this still left a minimum of 47 errors for every 1000 reference sites. LoFreq’s strand bias test successfully eliminated the single false positives originally identified in HCV run 1 and HCV run 2.

Figure 2 presents a detailed analysis of SNV calling by frequency for VarScan and ShoRAH, for SNVs with frequencies less than 15%. LoFreq results are excluded from this analysis, due to its near perfect precision for all data sets. Figure 2a shows that for the high diversity HIV data, perfect recall could be achieved for SNVs with population frequency greater than around 0.5%. Any reduction in recall due to the strand bias test was limited to SNVs with frequencies below this detection limit. Thus, although consideration of both recall and precision indicates the strand bias test does not improve on the raw HIV ShoRAH results, it is not significantly detrimental. The lack of improvement is due in part to the extremely high precision of 0.987 obtained by probabilistic clustering alone (see Table 3 and also Discussion).

For the HIV VarScan results, although the strand bias test did increase precision, more than 617 errors for every 1000 reference sites remained. Figure 2b indicates that this is the result of low frequency false positives. Restricting SNV calls to those covered by a minimum of 10 reads therefore rectified this problem to some extent (data not shown), however over 207 errors for every 1000 reference sites were retained. Recall was also adversely affected, due to the presence of very low frequency true SNVs in the HIV sample. Using a minimum read coverage of 10 had no effect on HIV ShoRAH results.

Figures 2b to 2d describe the number of false positives observed for each data set, binned in frequency increments of 1%. In general, ShoRAH calls less false positives than VarScan, particularly for very low frequency SNVs (*<* 1%). In most cases, the strand bias test was able to reduce or eliminate false positives across the range of frequencies observed (in Figure 2b-d, if the cross hairs are lower than the corresponding square or circle of the same colour, then the strand bias test reduced the false positive rate within the corresponding frequency bin). The effectiveness of the strand bias test was most notable for ShoRAH SNV calls from the moderate diversity HCV samples.

## Discussion

We have shown that SNV calling via probabilistic clustering is a powerful technique allowing detection of SNVs at frequencies lower than the corresponding sequencing error rate, as long as sample diversity is high (i.e., two or more true SNVs can be expected to occur within an observed read) and errors are random. When these conditions are not met, a statistical test of strand bias improves SNV calling precision.

The distribution of forward reads covering a true SNV at any one position could be expected to follow a binomial distribution, with mean value equal to the fraction of forward reads at that position multiplied by the total number of reads covering the variant. However, our results show that more accurate SNV calling is achieved if the expected number of forward reads is modelled using a beta-binomial read distribution, compared to either a binomial distribution or applying Fisher’s exact test.

Variation in the number of false positives between the HCV runs indicated error rates differed markedly, even though all samples were sequenced using Roche-454 Titanium technology. Notably, the ability to utilise empirical run specific error rates was one of the motivating factors behind LoFreq’s development. A similar result has been reported for Illumina sequencing as well, with individual runs sequenced on the same machine having dissimilar error profiles [11]. SNV calling precision after strand bias testing was relatively robust to choice of dispersion factor *σ*. Using a very low value of *σ*, however, compromised SNV recall, thus we would recommend choosing a value of *σ* closer to our upper estimate of 0.0111 when applying our test to real data.

For this reason, *σ* is set to 0.01 by default in our updated version of ShoRAH (*σ* is the only additional user specified parameter introduced to ShoRAH as a result of the strand bias test – all other parameters are estimated directly from the user’s data). For the HCV samples, which featured a moderate number of relatively high frequency true SNVs, the beta-binomial strand bias test provided a substantial reduction in error rates with no cost to true positive recall. This was true when applied to both the raw ShoRAH output and the raw VarScan output. However, both the relative reduction in error and absolute precision attained was greatest when the test was applied to the raw ShoRAH output. Unfortunately, it was not possible to apply our beta-binomial strand bias test to LoFreq’s raw SNV calls, as variant counts by forward and reverse read are not given in LoFreq’s output. LoFreq’s own Fisher’s exact test of strand bias successfully removed all false positives, however it also removed a number of true positives. Given the results reported here, we believe that a beta-binomial strand bias test may also remove these false positives, with substantially less cost to true positive recall.

For the HIV sample, the raw ShoRAH output gave the best results. This can be explained by the extremely strong performance of error correction via probabilistic clustering in this case. The ability to correctly identify true haplotypes via probabilistic clustering (and therefore the SNVs contained within the haplotypes) is a function of the frequency of the haplotypes, the number of variant positions within the haplotypes, and the error rate of the sequencing technology (see Methods). Whenever there is more than one true variant within a haplotype under consideration, it becomes possible to detect SNVs with population frequencies below the error rate of the sequencing technology. Consideration of HIV false positives called using VarScan indicates that locally, error rates may exceed 5%. For the HIV data, we achieved a detection limit well below this local error rate using ShoRAH alone, validating our argument. Figures 2a and 2b show that perfect accuracy (recall of one, with zero false positives) can be achieved by applying a detection limit of 3%, while a detection limit of only 0.5% gives perfect recall with only two false positives.

Another implication of our model is that accuracy should improve as the number of SNVs within a window of sites considered simultaneously increases, as demonstrated by the fact that ShoRAH performs better on the intrinsically high diversity HIV sample than on the moderate diversity HCV samples. Our argument is further supported by VarScan’s performance. Although for the HIV sample VarScan detected more true positives than ShoRAH, the corresponding extremely large number of low frequency false positives within the VarScan results suggests that this is the result of only considering one site at a time and indiscriminately calling low frequency SNVs below the associated detection limit (i.e., the local sequence error rate). LoFreq’s approach of discriminating SNVs individually by inferring an error probability based on the quality score assigned to individual nucleotides clearly overcomes this problem, although at the expense of recall.

While our strand bias test could not improve ShoRAH’s raw SNV calls for the HIV sample, we consider the slight reduction in recall with *σ* = 0.0111 (two additional very low frequency true SNVs were classified as errors) to be an acceptable cost, compared to the potential benefits of the test. It is usually not possible to know, a priori, how diverse a deep sequenced population may be. Even within highly diverse populations, genomic regions under purifying selection may have sub-optimal levels of diversity, allowing errors to appear clustered as true SNVs. Our strand bias test can help remove such errors, while leaving SNV calls in highly diverse regions relatively untouched. Our strand bias test could also be used to provide a level of confidence in results. If SNV calls are unaffected by applying a strand bias test, as in the case of our HIV sample, then it is likely that diversity was sufficient for probabilistic clustering to perform at its best.

Applying our strand bias test to raw VarScan SNV calls also improved precision in all cases. However, false positive SNV calls could not be reduced to a biologically useful level. The relative advantage of applying a strand bias test to ShoRAH’s output compared to VarScan’s output can be understood by considering how each of these programs identifies SNVs. VarScan distinguishes errors from true SNVs by examining the individual base quality scores of reads covering the potential SNV. VarScan is therefore reliant on accurate assignment of quality scores during the sequencing platform’s base calling pipeline. If an error nucleotide is falsely assigned a high quality score, then VarScan will retain it. Although LoFreq is also dependent on quality scores assigned by the sequencing platform, it has some resilience to this effect as quality information from all aligned nucleotides at a position is considered when making a call; errors at a given position would have to systematically be assigned high quality scores for LoFreq to incorrectly call an SNV. In contrast to VarScan, ShoRAH is independent of quality score information. ShoRAH works by removing any potential SNVs that are randomly distributed among reads, rather than clustering with nearby potential SNVs - regardless of whether they occurred during library preparation, PCR amplification, or read synthesis. Errors retained during clustering are likely to be systematic sequencing errors, which tend to exhibit a strand bias.

## Methods

### Statistical analysis of SNV calling based on probabilistic clustering

SNV calling from ShoRAH output involves parsing a set of ‘haplotypes’ for variants with respect to a reference sequence. These haplotypes are generated by deriving consensus sequences from groups of probabilistically clustered aligned reads. Because only the consensus sequence within each cluster is considered, SNVs will be correctly called, provided reads are correctly clustered so that the haplotypes can be identified from the centroids of the clusters.

The detection limit for true SNVs therefore depends on the detection limit for haplotypes. The detection limit for haplotypes is the haplotype frequency at which observed variants are equally likely to be errors (co-occurring within reads due to chance) or biological variants (corresponding to a true haplotype). For example, if two haplotypes differ at only one site (i.e., they have a Hamming distance of one), then the detection limit is intuitively equal to the error rate, *ϵ*. If, however, the haplotypes differ at two sites (with Hamming distance of two), then it takes two specific sequencing errors to mutate a read originating from one haplotype into a read that is equal to the other haplotype. This occurs with a probability of the order *ϵ*^2^. Now, *ϵ*^2^*< ϵ*, allowing haplotypes (and consequently SNVs) to be detected at frequencies lower than the error rate provided two sites are considered simultaneously (this has been exploited, for example, in [19]).

To investigate this property more formally, we study the probability of correctly assigning a read to either of two haplotypes: a minor haplotype with frequency *f* containing the same variants as the read under consideration, and a major haplotype (representing the next most similar pre-existing reconstructed haplotype) with frequency 1 − *f* . To simplify our analysis, we consider only those sites where these two haplotypes differ, as other sites are uninformative when choosing which haplotype to assign the read to.

According to this model, the major and minor haplotypes may be represented by the binary sequences 0 = 000 *. . .* 0 and 1 = 111 *. . .* 1 of length *d*, where *d* is the Hamming distance between these two haplotypes. Now, let the random variable *H* = (*H*_1_*, . . . , H*_*d*_) denote the true haplotype, such that *P*(*H* = 0) = 1 − *f* and *P*(*H* = 1) = *f* . Observation errors are assumed to occur independently with uniform per site probability *P*(*E*_*i*_ = 1) = *ϵ*, where each random variable *E*_*i*_ indicates the occurrence of an error at site *i*. The observed read, *Y*, is the true haplotype *H* subject to observation error: *Y*_*i*_ = 0 if (*H*_*i*_, *E*_*i*_) = (0*,* 0) or (1*,* 1), and *Y*_*i*_ = 1 otherwise. The full model is defined as follows:

(1) $$\begin{array}{l} H∼\;\mathrm{Bernoulli}\left(f\right),\;H\in \;\left\{\underline{0},\underline{1}\right\}\\ E_{i}∼\;\mathrm{Bernoulli}\left(\epsilon \right),\;E_{i}\in \;\left\{0,\;1\right\},\;i=1,...,d\\ Y_{i}∼\;H_{i}+E_{i}\;\mathrm{mod}2,\;i=1,...,d. \end{array}$$

In particular, *P* (*Y* = 1 | *H* = 0) = *ϵ*^*d*^ and *P* (*Y* = 1 | *H* = 1) = (1 − *ϵ*)^*d*^.

In order to assign the read correctly, we need to decide whether the observed read, *Y* = 1, is more likely to originate from the minor haplotype *H* = 1 without error or from the major haplotype *H* = 0 through (multiple) errors. This decision represents a crucial step in the iterative probabilistic assignment of reads to haplotypes within ShoRAH – if the constituent reads of a low frequency true haplotype are just as likely to originate from a higher frequency haplotype through sequencing errors, then they may be incorrectly assigned to the higher frequency haplotype. In this case, the low frequency haplotype falls below the limit of haplotype detection, and may not be represented in the final set of clustered reads.

Based on this model, the assignment will be correct if:

(2) $$P\left(H=\underline{0}|Y=\underline{1}\right)<P\left(H=\underline{1}|Y=\underline{1}\right).$$

Using Bayes’ theorem and Eqs. 1, this inequality becomes *ϵ*^*d*^(1 − *f* ) *<* (1 − *ϵ*)^*d*^*f*, or

(3) $$\epsilon \;<\;{\left[1+{\left(\frac{1-f}{f}\right)}^{1/d}\right]}^{\;-1}.$$

For noise levels *ϵ* below this upper bound, we would assign the reads correctly. For *d* = 1, the condition is *ϵ < f*, and for *ϵ* and *f* small, it is approximately *ϵ*^*d*^ *< f*. Thus for a given per nucleotide sequencing error rate, the frequency limit for haplotype detection decreases as the number of true SNVs observed within a read increases.

In reality, the process of haplotype inference is of course more complicated than presented here – our simplified argument is designed to merely demonstrate in a quantitative way that by observing the co-occurrence of variant sites, more power can be achieved when calling low frequency haplotypes (and hence SNVs) than by considering variant sites independently.

### Strand bias test

We assume that at any given genomic position, the number of forward reads carrying a variant follows a beta-binomial distribution. More formally, let *F*_*i*_ and *R*_*i*_, respectively, denote the total number of forward and reverse reads covering position *i*. The fraction of forward reads is *μ*_*i*_ = *F*_*i*_*/*(*F*_*i*_ + *R*_*i*_). We define *n*_*ib*_ as the total number of reads (forward and reverse) covering the variant base *b* at sequence position *i*. The number of forward reads carrying variant *b* at position *i* is modelled by the beta-binomial random variable *X*_*ib*_ ~ BetaBin(*n*_*ib*_, *μ*_*i*_, σ) such that 0 ≤ *X*_*ib*_ ≤ *n*_*ib*_ and

(4) $$\begin{array}{ll} P\left(X_{\mathrm{ib}}=x\right) & =\frac{\Gamma \left(n_{\mathrm{ib}}+1\right)}{\Gamma \left(x+1\right)\Gamma \left(n_{\mathrm{ib}}-x+1\right)}\\ & \;\times \frac{\Gamma \left(1/\sigma \right)\Gamma \left(x+\mu_{i}/\sigma \right)\Gamma \left(n_{\mathrm{ib}}+\left(1-\mu_{i}\right)/\sigma -x\right)}{\Gamma \left(n_{\mathrm{ib}}+1/\sigma \right)\Gamma \left(\mu_{i}/\sigma \right)\Gamma \left(\left(1-\mu_{i}\right)/\sigma \right)} \end{array}$$

The expected value of *X*_*ib*_ is E(*X*_*ib*_) = *n*_*ib*_*μ*_*i*_, and *σ* is a dispersion parameter, defined such that

(5) $$\mathrm{Var}\left(X_{\mathrm{ib}}\right)=n_{\mathrm{ib}}\mu_{i}\left(1-\mu_{i}\right)\left[1+\frac{\sigma \left(n_{\mathrm{ib}}-1\right)}{\left(1+\sigma \right)}\right].$$

The dispersion parameter *σ* determines the spread of the distribution. We assume that, unlike *n*_*ib*_ and *μ*_*i*_, *σ* does not change between each variant within a sample. Both *n*_*ib*_ and *μ*_*i*_ can be easily estimated directly from the data for each individual potential SNV under question. We employed ML estimation to obtain *σ* estimates for each sample, by considering the *K* true SNVs $x_{i_{1}b_{1}},\ldots ,x_{i_{k}b_{k}}$. The likelihood function is:

(6) $$L\left(\sigma \right)=\prod_{j=1}^{K}P\left(X_{i_{j}b_{j}}=x_{i}{{}_{{}_{j}b}}_{{}_{j}}\right).$$

ML estimation was performed in R with the package mle2, using the default Nelder and Mead optimisation method and a start value of one. (Per-sample ML estimation of *σ* was only possible as this study employed control data with known SNVs. For real data, we suggest using a *σ* of around 0*.*01, close to our highest estimate of *σ* from our control data. Our analysis indicated that results are fairly stable with respect to *σ*).

Our null hypothesis, *H*_0_, is that for any given true SNV, the number of forward reads follows a beta-binomial distribution with parameters *μ*, *n*, and *σ*. The null hypothesis *H*_0_ is rejected if, under *H*_0_, the probability to observe data as extreme or more extreme than that observed is smaller than a threshold *p*. That is, given an observation *x*_*ib*_, we reject *H*_0_ if min {Φ(*x*_*ib*_)*,* 1 − Φ (*x*_*ib*_)} × 2 *< p*, where Φ(*x*_*ib*_) = *P*(*X*_*ib*_ ≤ *x*_*ib*_) is the beta-binomial cumulative distribution function (calculated in R using the gamlss beta-binomial CDF), with parameters estimated as described above.

If, for a potential SNV, the null hypothesis is rejected, then the potential SNV is considered to be an error. As we do not care which direction the strand bias occurs in, a two-sided test was performed by multiplying the smallest tail of Φ(*X*_*ib*_) by two. We then applied the Benjamini-Hochberg correction for multiple testing, giving the final p-value *p*. If *p <* 0*.*05, we reject the potential variant as an error.

To investigate the effect of *σ* on the strand bias test (in particular the possibility of overfitting), strand bias tests with each estimate of *σ* were applied to SNV calling results obtained from ShoRAH and VarScan for all data sets. A test with *σ* = 0 was also performed, which models a binomial forward read distribution. For comparison, two-sided Fisher’s exact tests were also applied, based on counts of the forward and reverse reads, again with a Benjamini-Hochberg correction for multiple testing. It was not possible to apply our strand bias test to SNV calls from LoFreq, as LoFreq’s output does not record the number of forward and reverse reads covering a variant. LoFreq does however report a p-value based on a Fisher’s exact test of strand bias. Thus we were able to report the results of a Fisher’s exact test of strand bias applied to raw LoFreq SNV calls, again applying a Benjamini-Hochberg correction for multiple testing within R and rejecting any variants for which *p <* 0*.*05.

To further compare the performance of Fisher’s exact test, a binomial strand bias test, and our beta-binomial test (with *σ* = 0*.*0111), precision recall curves were constructed within R for each test applied to ShoRAH or VarScan SNV calls from HCV run 1 and HCV run 2. This was done by calculating the precision and recall for p-value cut offs varying between zero and one (in increments of 0.005) and plotting the results.

### Sample sequencing, alignment, and frequencies

For HCV E1/E2 samples, a mixture of four different 2324bp fragments (excluding primer regions), each covering the 3′ end of the HCV core to the 5′ end of the p7 region was cloned into the pGEM-T Easy vector and sequenced using Roche-454 FLX Titanium as described previously (HCV run 1) [6].

To investigate variation in *σ* between sequencing runs, a second round of Roche-454 FLX Titanium sequencing was performed on this same library of HCV clone mixtures (HCV run 2). For the HIV gag/pol-gene fragments, 10 PCR products from subtype-B clinical isolates were propagated within the pCRII-TOPO vector, excised, and subjected to a single round PCR before Roche-454 FLX Titanium sequencing, as previously described [22].

Prior to alignment, reads were trimmed using a sliding window of 8nt. The trim was made at the start of the first 8nt window from either end with an average quality greater than 20. For all samples, alignment was performed using bwasw [28], with default parameters. For the HCV samples, alignment was performed against the entire pGEM genome with an HCV 1a strain insert, corresponding to the inserted fragments [6]. For the HIV sample, alignment was performed against the appropriate region of the HXB2 HIV reference genome, from base 2253 to 3497.

To estimate the true frequency of each fragment within the mixture, the known sequences of each fragment were used to locate SNV positions uniquely identifying each fragment. Fragment frequencies were then calculated by averaging the frequency of all SNVs unique to a fragment haplotype, then normalising the results so that the sum of all haplotype frequencies was one.

### SNV calling

ShoRAH 0.6 was run on all three alignments described above. For each run, *α* was set to 0.01, window size was set to 360 with a step size of 120, and the number of iterations to 15 times the window coverage for each window. To call an SNV from the raw ShoRAH output, locally reconstructed windows were parsed for variant positions. For an SNV to be called, the same variant was required to be found in at least two of three overlapping windows, with a posterior probability greater than 0.9.

This approach was compared to single site SNV calling, as implemented in LoFreq and VarScan. LoFreq is a single site variant caller that uses per nucleotide quality score information to inform a statistical test for each potential SNV. LoFreq is tailored to calling low frequency variants in deep sequencing data, and is thus ideally suited to identifying variants in viral populations. SNV calling was performed using the script lofreq_snpcaller.py (v0.6.0) with default options. VarScan was also employed due to its ability to report the required read statistics for the strand bias test, and its single site mode of SNV calling. However, we acknowledge that VarScan is intended for use on diploid resequencing data rather than deep sequencing of viral populations, and do not imply that results reported here are indicative of performance under normal usage conditions. VarScan’s pileup2snp command (v2.2.3) was used to call SNVs. Default values for quality filtering were used. Minimum variant frequency was set to zero, to allow comparison with ShoRAH. For all variant callers employed, we limited called variants to those covered by more than one read. To allow a fair comparison, SNV calling using LoFreq and VarScan was only attempted in areas of the genome covered by three ShoRAH windows. This corresponds to positions 18 to 2324 of the HCV fragments (2307 bp in total), and positions 240 to 1005 of the HIV fragments (766 bp in total). In total, 38 true SNVs were analysed for the HCV samples, and 188 true SNVs were analysed for the HIV sample.

An attempt to call SNVs using V-Phaser was made, as this program combines joint SNV calling with quality score based methods. However, the program crashed when used on any of our samples, possibly due to the high coverage of samples. Correspondence with the authors did not resolve the issue, thus the comparison was not able to be made. SNV calling was also attempted using an empirical k-mer based algorithm (‘Kec’) [23]. After running for more than 150 cpu hours, this software did not produce a result, at which point this comparison was also abandoned. SNV calling with DeepSNV [25] and using the approach described by Flaherty *et al.*[18] was not attempted due to the lack of control or multiple samples.

For ShoRAH and VarScan, raw results, as well as results subjected to our strand bias test, are reported. For the purposes of this paper, all strand bias tests were performed in R as described above. This was done to facilitate an exact comparison between VarScan and ShoRAH results. However, in conjunction with this manuscript we have released a new version 0.6 of ShoRAH with integrated SNV calling and strand bias testing, which does not require R.

## Conclusions

We have demonstrated that by combining probabilistic clustering with a statistical test of strand bias, highly accurate SNV calling can be achieved. This is because the strengths and weaknesses of these two methods are complementary: probabilistic clustering relies on errors occurring randomly and independently, while strand bias tests detect systematic errors with atypical read direction distributions.

Using a statistical model and through analysis of control data we investigated the conditions under which probabilistic clustering can be used to call SNVs. We show that the limit of SNV detection is *ϵ*^*d*^ *< f*, where *ϵ* is the rate of randomly occurring sequencing errors, *d* is the number of true variants observed within a read length, and *f* is the frequency of the haplotype to which the variants belong. In general, the SNV detection limit decreases as true diversity increases. This is consistent with our empirical analysis of SNV calling from control data with various diversity levels. Importantly, for SNV calling via probabilistic clustering to have an advantage over approaches that consider individual variants one at a time, more than one true variant must be contained within a read length.

To insure against situations where diversity is inadequate or errors are non-random, we suggest a strand bias test be applied to SNV calls generated via probabilistic clustering. The test we propose uses a beta-binomial model of forward read distribution for true SNVs. By comparing our test to the performance of either Fisher’s exact test or a test based on a binomial model of forward read distribution, we show that the forward read ratio of true SNVs is overdispersed. Our analysis indicates that as long as this overdispersion is taken into account, a statistical test of strand bias will not affect recall. This means such a test can be safely applied and will not adversely affect accuracy, even in situations where no improvement in precision is obtained.

While our analysis is presented in the context of SNV calling using ShoRAH, the insights into SNV calling via probabilistic clustering should be applicable to any method that considers multiple variant sites simultaneously. Furthermore, our strand bias test has the potential to improve SNV calling precision for any variant detection pipeline.

## Availability

The SNV caller and strand bias test have been integrated into ShoRAH 0.6, available at http://www.cbgethz.ch/software/shorah. Haplotypes, aligned reads, and reference sequences used in this study may be obtained from https://wiki-bsse.ethz.ch/display/ShoRAH/Data. Contact: shorah@bsse.ethz.ch.

## Competing interests

The authors declare that they have no competing interest.

## Authors’ contributions

KM implemented the strand bias test, analysed control data, and in conjunction with OZ developed the software ShoRAH v0.6. NB developed the SNV calling model. KM wrote the manuscript, with editing by FL, OZ, and NB. NB and FL conceived of the overall study. RB and OZ provided control data. All authors read and approved the final manuscript.

## Supplementary Material

Additional file 1 **Tab delimited list of true SNVs for HCV haplotypes.** Format: Position \t Reference nucleotide \t Variant nucleotide \t Frequency run 1 \t Frequency run 2. To facilitate analysis with supplied reference sequences and alignments, position is given with respect to the start of the vector; subtract 222 to get position with respect to the HCV fragment. Frequencies are calculated based on the estimated true frequency of haplotypes (Table 1). Click here for file

Additional file 2 **Tab delimited list of true SNVs for HIV haplotypes.** Format: Position \t Reference nucleotide \t Variant nucleotide \t Frequency. Frequencies are calculated based on the estimated true frequency of haplotypes (Table 2). Click here for file
